# The relationship amid land finance and economic growth with the mediating role of housing prices in China

**DOI:** 10.3389/fpsyg.2022.976236

**Published:** 2022-08-17

**Authors:** Qian Sun, Yan Feng, Yong Tang, Wen Kuang, Sohail Ahmad Javeed

**Affiliations:** ^1^Management School, Hunan City University, Yiyang, China; ^2^Hunan New Urbanization Research Institute, Yiyang, China

**Keywords:** land finance, economic growth, housing prices, prefecture level cities, mediating role

## Abstract

Taking cities at prefecture-level and above in China as the research object, we theoretically analyze the effects of land finance on housing prices and economic growth as well as the effects of housing prices on economic growth, consider the mediating role of housing prices, and construct a random-effects model of land finance affecting economic growth. It is of great significance to make rational use of land finance to promote the economic development and formulate the “city specific policies” plan for the real estate market. The 278 resource-based cities with relatively well-developed land and real estate markets in China are selected to test the mediating effect of housing prices on land finance affecting economic growth in resource-based cities by type based on panel data from 2011–2019. The results show that (1) land finance significantly and positively affects economic growth and housing prices in cities at the prefecture-level and above nationwide, but there is some variability in the degree of influence. The central region has the smallest impact on economic growth but the largest impact on housing prices; the eastern region has the deepest impact on economic growth; and the western region has the smallest impact on housing prices. (2) In the national sample cities and cities in the northeast region, housing prices have a significant partial mediating effect at the 1% level on economic growth affected by land finance, accounting for 22.03 and 2.84%, respectively. The mediating effect of urban housing prices on land finance affects economic growth in the eastern, central, and western regions is not significant.

## Introduction

After the implementation of the tax-sharing reform in China in 1994, the central government received financial power, but the local government has not changed its power accordingly. The mismatch of rights and responsibilities has put local governments under tremendous pressure, even to the extent that their revenues do not cover their expenditures. To compensate for the financial pressure caused by the mismatch between financial rights and responsibilities, local governments have made efforts to seek extra-budgetary funds. So in this vein, land financing has huge importance because land finance is the name given to a transaction that provides funds for the acquisition, expansion, or improvement of land but excludes a construction loan or a mortgage loan (Mo, 2018).

Land finance, centered on land concessions, has become the main way for local governments to generate money. Therefore, revenue from the concession of state-owned land-use rights was 8,750.1 billion yuan from 2021 and accounting for 78.37% of local general public budget revenue. The total amount of land concessions in 300 cities nationwide is 5.9 trillion yuan, and 33 cities have land concessions of more than 50 billion yuan. In this context, Land concessions provide major funding for the transformation and upgrading of urban industrial structures, infrastructure construction, and livelihood welfare spending. Local governments' land finance has not only bridged the fiscal gap to some extent but also played an important role in the rapid economic development (Zhao et al., 2017).

In addition, the over-reliance of local governments on land finance also has a series of negative effects. The central government's assessment of local officials is a tournament model (Zhou, 2007), and GDP and local finance have become important indicators for performance assessment and promotion. Local governments use the asset value attribute of land to promote enterprise development by suppressing the rise of land prices for industrial land on the one hand and to finance infrastructure construction and meet the needs of urbanization construction and local production and operation by expanding the revenue from residential land concessions on the other.

Therefore, local governments have a strong incentive to push up housing prices (Rao and Ge, 2014). It is widely believed that land finance is one of the main reasons for the rapid rise in housing prices (Wu and Ou, 2014). The rapid rise in housing prices has brought about a series of problems such as the “de-realization” of the economy and the widening gap between the rich and the poor. In addition, over-reliance on land finance can easily induce rent-seeking and corruption among officials, exacerbate financial risks, and lead to the hollowing out of industries, etc.

Although, regarding the research on the impact of land finance on economic growth, scholars' views are somewhat divergent. Some scholars believe that land finance relieves local fiscal pressure to a greater extent and has a positive driving effect on economic growth, government activism, investment in fixed assets, industrial restructuring (Xia et al., 2014), labor force transfer from the traditional agricultural sector to modern industrial and manufacturing sectors (Yue and Lu, 2016), and population agglomeration effect from labor mobility (Lu and Teng, 2020), etc. become important transmission mechanisms for land finance to influence economic growth.

Moreover, some scholars also believe that there is a Kuznets inverted U-shaped curve between land finance and economic growth, that is, land finance is beneficial to economic growth in the short term, but over-reliance on land finance will inhibit economic growth (Wu and Wang, 2017; Hu and Liu, 2020). In the long run, land finance ignores the balanced development of secondary and tertiary industries and the effective allocation of resources (Zou and Liu, 2015), and the economic growth model driven by fixed-asset investment is unsustainable (Cai et al., 2017), and even produces an early deindustrialization effect (Zhou and Zhou, 2018).

Besides, regarding the relationship between land finance and housing prices, a large number of studies have found a positive effect of land finance on housing prices. The fiscal imbalance of local governments caused by the tax-sharing reform makes local governments overly dependent on land concessions, and land finance is an institutional factor that drives up house prices (Gong, 2012; Wang and Wu, 2019).

Regarding the impact of housing prices on economic growth, it is generally believed that “crowding-in” and “crowding-out” effects exist simultaneously (Yuan and Yuan, 2019). On the one hand, under the effect of wealth and collateral utility, rising house prices increase financing capacity, increase household wealth, and promote consumption and investment (Chaney et al., 2012; Jack et al., 2017).

In summary, academics generally agree that land finance significantly affects economic growth directly or indirectly, with industrial restructuring, urbanization, and population mobility as important mediating variables. Since land finance originates from local governments raising residential land prices, land finance leads to higher housing prices. There is also a significant effect of housing price on economic growth under multiple effects such as wealth effect and collateral effect. As can be seen, in the studies on the relationship between land finance, housing prices and economic growth, academics have not placed land finance, housing prices and economic growth under the same framework to study the role of housing prices in the relationship between land finance and economic growth. Therefore, the research contribution of this paper is that focuses on the mediating effect of housing price on land finance affecting economic growth using the mediating effect model, which improves the research content of land finance affecting economic growth with a more focused research perspective.

The essence of land finance is that local governments obtain land revenue by granting state-owned land use rights. On the one hand, local governments can use land finance to increase investment in infrastructure fixed assets, drive investment in local industrial enterprises, reduce land costs for enterprises, promote enterprise development and achieve economic growth. On the other hand, land finance can be used for infrastructure construction and land resource allocation to guide investment, promote the development of secondary industry to tertiary industry and the transformation of low value-added industry to high value-added industry, and promote economic growth.

Land finance is rooted in the high price of residential land for sale, which leads to higher housing prices and thus affects economic growth. Thus, housing prices mediate the impact of land finance on economic growth. On the one hand, the rise in housing prices driven by land finance will lead to higher wages and lower profit margins for enterprises, which will lead to a “de-realization” of capital flows and thus inhibit economic growth. On the other hand, the rise in housing prices driven by land finance will further promote real estate investment and boost the upstream and downstream industrial chains of real estate, thus promoting economic growth.

**Figure 1:**
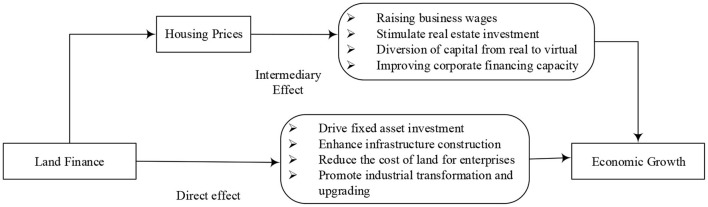
The theoretical model of land finance, housing price, and economic growth.

In summary, this paper puts land finance, housing price and economic growth in the same framework, and study the role of housing price in land finance and economic growth. It proposes that urban land finance will promote economic growth and housing prices will mediate the effect of land finance on economic growth. The panel data of prefecture-level and above cities from 2011 to 2019 are also used to construct a mediating effect model, and the stepwise regression method and Bootstrap method are used to empirically test the effects of land finance on economy. It is of great significance to make rational use of land finance to promote the economic development and formulate the “city specific policies” plan for the real estate market. The remaining part of this paper has been classified into various sections. Section Literature review shed light on literature review and hypothesis development. Section Materials and methods reveals the model settings. Section Results and discussion presents the analysis and discussion of the results. Section Conclusion and policy implications shed light on the findings of this study and recommendations. Figure 1 shows the theoretical model of this paper.

## Literature review

Regarding the research on the impact of land finance on economic growth, scholars usually believe that in the short term, land finance relieves local financial pressure to a greater extent, provides financial support for the transformation and upgrading of industrial structure, urban infrastructure construction and improvement of people's welfare, and has a positive effect on economic growth. The central government's tournament model for assessing local officials has boosted local governments' enthusiasm to expand land finance, drive investment in fixed assets and increase infrastructure construction, directly promoting economic growth (Zhou, 2007; Cai et al., 2017).

In the long run, the economic growth model driven by fixed asset investment is unsustainable because land finance neglects the balanced development of secondary and tertiary industries and the effective allocation of resources (Zou and Liu, 2015). Therefore, many scholars believe that there is a Kuznets inverted U-shaped curve between land finance and economic growth, i.e., land finance is beneficial to economic growth in the short term, but over-reliance on land finance will inhibit economic growth (Wu and Wang, 2017; Hu and Liu, 2020).

Land finance indirectly affects economic growth through mediating factors such as industrial restructuring (Xia et al., 2014), labor migration from traditional agricultural sectors to modern industrial and manufacturing sector (Yue and Lu, 2016), and population agglomeration effects from labor mobility (Lu and Teng, 2020). Moreover, Zhou and Zhou (2018) point out that land finance ostensibly promotes economic growth through industrial restructuring, but the utility of induced early deindustrialization undermines economic growth potential in the long run (Lv and Liu, 2012).

In addition, Guo and Zhou (2020) focus on the mediating effect of urbanization and industrial structure upgrading on the impact of land finance on economic growth, pointing out that industrial structure upgrading enhances the enhancing effect of land finance on economic growth; after urbanization reaches a certain level, the positive effect of land finance on economic growth weakens, especially in the late stage of high urbanization, the expansion of land finance will inhibit economic growth (Li and Hua, 2018). Rent-seeking due to scarcity of land resources can squeeze production and distort land finance allocation, leading to a negative correlation between land finance and economic growth (Xue and Chi, 2010; Liu and Chen, 2015).

***H1: Land Financing and Economic Growth are significantly connected***

Regarding the relationship between land finance and housing prices, numerous studies have found a positive effect of land finance on housing prices. The tax sharing reform has led to fiscal imbalance of local governments, and land finance has become an institutional factor driving up housing prices (Diao, 2015; Wang and Wu, 2019). After controlling for other factors affecting housing prices, the more dependent local governments are on land finance, the faster urban housing prices rise (Zhang and Li, 2010; Wang and Gao, 2011). Other factors can reinforce the influence of land finance on housing prices. Guo (2013) developed a multivariate model of house prices in China from 1999–2009, and found through static and dynamic panel analysis that there are mutual feedback effects among land finance dependence, fiscal gap and house prices.

In addition, Tang and Ma (2017) and Xu et al. (2020) placed fiscal pressure, land finance and housing prices in the same research framework and found that fiscal pressure, as an institutional factor, has an indirect effect on house prices through land finance, thus solidifying the “ratchet effect” on house prices. In addition, there are regional differences in the impact of land finance on economic growth and housing prices due to differences in land use efficiency (Zhang and Weng, 2022), imitation effects of local government policy making, and the location fixity of housing (Zou, 2016; Li and Hua, 2018).

***H2: Land Financing and Housing Prices are significantly connected***

Regarding the impact of housing prices on economic growth, it is generally accepted that there are both “crowding-in” and “crowding-out” effects (Yuan and Yuan, 2019). On the one hand, with the effect of wealth and collateral, higher housing prices increase the ability to finance, increase household wealth, and promote gconsumption and investment (Chaney et al., 2012; Jack et al., 2017). For example, Chirinko et al. (2008) find that a 1.5 percentage point increase in house prices is associated with a 0.4 percentage point increase in GDP; static and dynamic analyses by Ren and Chen (2019) show a significant interaction between house prices and economic growth (Binkai et al., 2018).

On the other hand, some scholars argue that rising house prices lead to lower real income levels, reduce the consumption incentives of those in immediate need of housing (Zhao and Zhang, 2016), push up corporate labor costs, reduce the profitability of industrial enterprises, and thus inhibit real economic growth (Chen et al., 2018). Also, Gelatin et al. (2013) argue that excessive increases in house prices that deviate from the macro economy affect the response of firms and investors to price signals, leading to a failure to allocate capital rationally and adversely affecting the economy. This will have a negative impact on the economy (Shen and Dong, 2018). Furthermore, Shen and Dong (2018) argue that the effect of house price volatility on economic growth shows significant investment size heterogeneity. Huang and Ni (2020) empirically analyze that there is an inverted U-shaped relationship between rising house prices and economic growth (Huang and Ni, 2020).

***H3: Land Financing has effects on Economic Growth with the Mediating Role of*** ***Housing Prices***

## Materials and methods

### Model setting

In order this paper focuses on two major relationships, namely, the effect of land finance on the economic growth of resource-based cities and the effect of land finance on the economic growth of resource-based cities through housing prices. To this end, a mediating effect model needs to be constructed to test the mediating effect of housing prices on the impact of land finance on economic growth in resource-based cities. Currently, there are three main single tests for mediating effects: the stepwise test regression coefficient method first proposed by Baron and Kenny (1986), the coefficient product test (Sobel, 1982), and the coefficient difference test (Freedman and Schatzkin, 1992). It should be noted that although each of these three methods has advantages and disadvantages in terms of statistical test errors and test efficacy, the applicability of using a single method for testing is relatively low Mackinnond et al. (2002). For this reason, this paper combines the Bootstrap method proposed by Preacher and Hayes (2008) with the new mediation test process constructed by Wen and Ye (2014) for testing mediation effects.

Specifically, the baseline model is first constructed to do the baseline regression, i.e., the regression without considering the housing price variables. Since the study subjects are resource-based cities, which are large in number and have strong individual heterogeneity, it is necessary to control the city individual effect in the model to control the problem of omitted variables due to individual city changes. In addition, considering the time trend problem caused by the change in urban economic growth over time, the time trend effect needs to be controlled in the model. The baseline model is shown in Equation (1).


(1) $$\begin{array}{l} ecog_{it}=\alpha_{0}+\alpha_{1}landfin_{it}+\alpha_{2}control_{it}+\alpha_{3}charac_{it}\\ \;+\mu_{i}+\alpha_{4}t+\varepsilon_{it} \end{array}$$

Next, the mediation model as shown in Equation (2) is constructed to carry out the regression of the mediating variables.


(2) $$\begin{array}{l} prihou_{it}=\beta_{0}+\beta_{1}landfi_{it}+\mu_{i}+\beta_{2}t+\varepsilon_{it} \end{array}$$

Finally, a model introducing the mediating variable housing price [as in Equation (3)] is constructed to carry out the overall regression.


(3) $$\begin{array}{l} ecog_{it}={\alpha^{\prime }}_{0}+\gamma_{1}landfin_{it}+\gamma_{2}prihou_{it}+{\alpha^{\prime }}_{2}control_{it}\\ \;+{\alpha^{\prime }}_{3}charac_{it}+\mu_{i}+\alpha_{4}t+\varepsilon_{it} \end{array}$$

In the above equations: is the number of cities; *t* is the year; α, β, and γ are the regression coefficients of different model variables; is the individual effect; and is the time trend. In addition, the model introduces *control*_*it*_ control variables and *charac*_*it*_ characteristic variables to solve the omitted variable problem and control the attribute characteristics of different cities.

**Figure 2:**
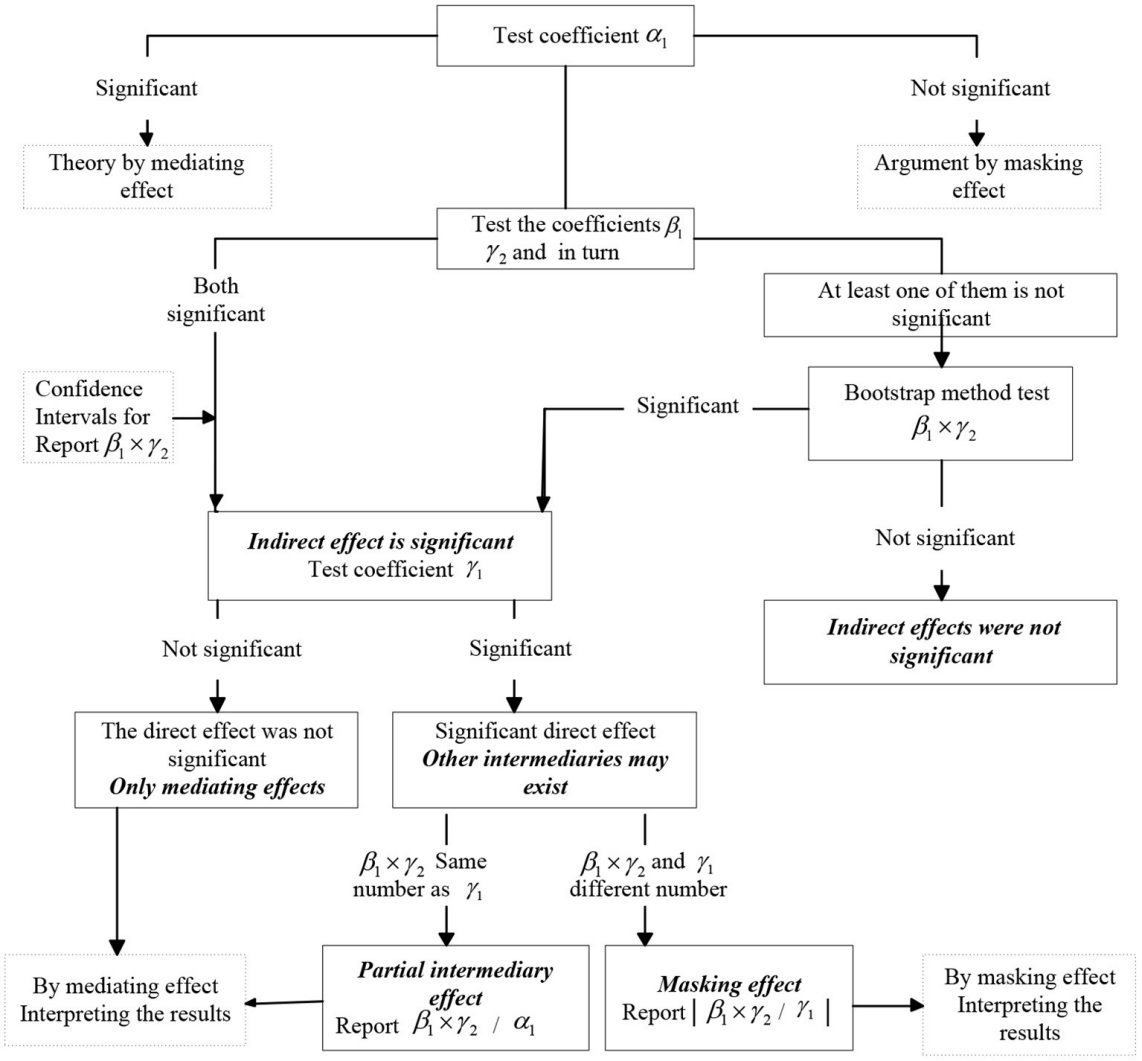
Mediation effect testing process.

The judgment process is shown in Figure 2: ➀ If α_1_ is significant, it means that there is a total effect of land finance on economic growth and it is a mediating effect, otherwise it is a masking effect. ➁ If β_1_ is significant, it means that land finance affects housing price. ➂ If γ_2_ is significant, it means that housing price affects economic growth. ➃ If at least one of β_1_ and γ_2_ is insignificant, bootstrap method needs to be introduced to test the significance of β_1_ × γ_2_. If it is significant, it means that the indirect effect of land finance affects economic growth is significant; if it is not significant, it means that the indirect effect is not significant and the test can be stopped. ➄ If β_1_ and are significant at the same time or β_1_ × γ_2_ is significant, then we need to pay attention to whetherγ_1_ is significant or not, and γ_1_ is the direct effect of land finance on economic growth. If γ_1_ is not significant, it means that housing price plays a fully mediating effect; if γ_1_ is significant, it means that housing price plays a partial mediating effect; if β_1_× γ_2_ and γ_1_ have the same sign, the mediating effect accounts for; if β_1_ × γ_2_ and γ_1_ have different signs, housing price plays a masking effect and the effect accounts for |β_1_ × γ_2_/γ_1_|.

### Variable selection and data sources

#### Explained variable

Economic growth (*ecog*). Local economic growth is generally measured by the size of GDP per capita or economic growth rate indicators, including both GDP per capita at comparable prices and GDP per capita at current year prices. Considering that the selected explanatory and control variables are mostly measured in current year prices, GDP per capita at current year prices is used as an indicator of regional economic growth.

#### Core explanatory variable

Land finance (*landfin*). Land finance includes not only land concession revenue, but also land-related real estate taxes and bank proceeds obtained through mortgaged land. Referring to other studies at [39,40], land concession revenue is used as a proxy variable for land finance, considering that land concession revenue accounts for the largest share of land finance and that economic growth is characterized by per capita averages. Later, the proportion of land concessions to the size of the general public budget is used as a proxy variable for land finance to conduct robustness tests.

#### Mediating variable

Housing price (*prihou*). Drawing on Li et al. (2020) and Zhan et al. (2020) and others, the average urban residential sales price was chosen as a proxy variable for housing prices.

#### Control variables

To reduce the possibility of endogeneity problems and to consider robustness, based on the economic growth theory and the research of Hu and Liu (2020), firstly, fiscal pressure, year-end population, research and experimental development funding, government size, foreign direct investment, real estate investment size, unemployment rate, income level of the population, and technological innovation were initially selected as control variables. These control variables are then tested by VIF (Variance Inflation Factor) to eliminate those variables with values greater than 10 that have multiplicative covariance problems, and finally, three control variables are selected: government size, industrialization level, and unemployment rate. Among them,

➀ Government Scale (*scalegov*): the government size is measured by the proportion of local government general public budget expenditure to GDP; ➁ real estate investment scale (*scalehou*): the real estate investment scale is measured by the proportion of real estate investment to GDP; ➂ resident income level (*incomeper*): the resident income level is expressed by the disposable income of urban residents in the current year.

#### Characteristic variables

To control for different city attribute characteristics as much as possible, drawing on practices such as Li et al. (2020), city characteristic variables are considered: industrialization level (*indus*): the level of industrialization is measured by the value of the secondary industry as a proportion of GDP for local governments; the level of infrastructure development (*infras*), using administrative districts.

Road area per capita in the domain is expressed; unemployment rate (*unemploy*): unemployment rate is measured by dividing the number of registered urban unemployed at the end of the year in each city by the value of the sum of the number of registered employed and unemployed at the end of the year. The above non-ratio variables are treated as natural logarithms in the regression equations. The variables involved and their measures are shown in Table 1.

**Table 1:** Variables declarations.

**Variable type**	**Variable name**	**Variable code**	**Definition and metric**
Explained variables	Economic growth	*ecog*	Logarithmic gdp per capita in current year prices
Explanatory variables	Land finance		Land premiums per capita for logarithmic combing
Mediating variables	Housing prices	*prihou*	Average urban residential sales price for logarithmization
Control variables	Government size		General public budget expenditure/GDP
	Scale of real estate investment	*scalehou*	Real estate investment/GDPP
	Income level of residents	*incomeper*	Urban residents' disposable income for the year is logarized
Characteristic variables	Infrastructure construction level	*infras*	Logging of road area per capita in the administrative area
	Industrialization level		Value of secondary industry/GDP
	Unemployment rate	*unemploy*	Number of unemployed at the end of the year/(number of employed at the end of the year + number of unemployed at the end of the year)

As of the end of 2020, the National Bureau of Statistics identified 297 cities at the prefecture-level and above nationwide. In this paper, 278 prefecture-level and above cities with relatively perfect land and real estate markets are selected for the study based on data availability, including 34 cities in the eastern region, 86 cities in the central region, 80 cities in the western region, and 78 cities in the northeastern region, accounting for 12.2, 30.9, 28.8, and 28.1%, respectively.

Urban housing prices are obtained from the national average urban residential sales price data monitored by the Xi Tai database. Since the domestic urban housing price monitoring database was built late and there are many missing data before 2010, the study period was selected from 2011–2019. The data on land premiums were obtained from the China Land and Resources Statistical Yearbook and the China wind database, respectively. Since the China Land and Resources Statistical Yearbook was not updated after 2018, the wind database has more missing data, and there are some differences in the statistical caliber of the two databases, the data of land concessions for 2018 and 2019 are based on the data of the China Land and Resources Statistical Yearbook from 2015–2017 using the AR model to estimate the relative coefficients, and then the wind database is The rest of the data are obtained from the China Urban Statistics Yearbook. The rest of the data were obtained from the China Urban Statistical Yearbook. The extreme values were winsorize, and individual missing data were supplemented by interpolation.

## Results and discussion

### Descriptive statistics

The results of descriptive statistics of the main variables are shown in Table 2. As can be seen from the table: the average urban housing sales price in China showed a year-by-year increase from 2011–2019, especially after 2016, the rate of increase accelerated significantly. Urban GDP per capita increased year by year from 2011–2017, and began to decline significantly in 2018 and 2019. Land premium per capita has been oscillating up and down from 2011–2019, and started to fall back in 2013 to 2017 and started to rise again year by year.

**Table 2:** Descriptive statistics of main variables.

**Variables**	**Statistical quantities**	**2011**	**2012**	**2013**	**2014**	**2015**	**2016**	**2017**	**2018**	**2019**
*ecog*	Mean	10.399	10.509	10.585	10.662	10.703	10.753	11.022	10.883	10.925
	S.D.	0.559	0.547	0.674	0.53	0.527	0.52	0.544	0.529	0.523
	Min	8.773	9.007	9.037	9.227	9.304	9.384	9.287	9.446	9.599
	Max	12.002	12.115	13.056	12.207	12.241	12.281	12.792	12.165	12.223
	Mean	7.211	7.212	7.594	7.337	7.047	7.059	7.421	7.713	7.829
	S.D.	1.078	0.944	0.959	0.904	1.048	1.137	1.171	1.084	1.055
	Min	2.816	4.261	4.965	3.956	4.012	2.816	4.122	4.651	4.155
	Max	9.783	9.536	10.339	9.973	9.83	10.204	10.406	10.429	10.51
*prihou*	Mean	8.519	8.5	8.557	8.576	8.558	8.601	8.776	8.915	8.947
	S.D.	0.445	0.413	0.418	0.414	0.422	0.458	0.499	0.51	0.503
	Min	7.582	7.813	7.916	7.925	7.952	7.847	7.709	7.733	7.714
	Max	10.368	10.189	10.487	10.528	10.512	10.781	11.073	11.062	11.073

### Unit root test for panel data

Before estimating the model, the data of each variable needs to be tested for smoothness to avoid the pseudo-regression problem. The research sample of this paper is the data of 278 prefecture-level and above cities in China from 2011–2019, which is short panel data, so the HT test method is selected for the unit root test of panel data, and the results are shown in Table 3. It can be seen that all variables significantly pass the smoothness test, and can be identified as a smooth series without pseudo-regression problems.

**Table 3:** Unit root test results.

**Variables**	**HT inspection**	**Results**
*ecog*	−20.4066^***^	Smooth and stable
*landfin*	−12.3902^***^	Smooth and stable
*prihou*	−8.0772^***^	Smooth and stable
*scalegov*	−24.4208^***^	Smooth and stable
*scalehou*	−20.5119^***^	Smooth and stable
*incomeper*	−12.3777^***^	Smooth and stable
*infras*	−29.1669^***^	Smooth and stable
*indus*	−8.6763^***^	Smooth and stable
*unemploy*	−15.4184^***^	Smooth and stable

*** *indicate significant correlation at the 10, 5, and 1% levels, respectively*.

### Analysis of regression results

In this paper, the models were estimated using Stata 15.0 software. To select the appropriate panel regression model, the Hausman (Hausman) test was applied to each model to determine whether to use fixed-effects regression or random-effects regression. The original hypothesis of the Hausman test was to use random effects regression, and the test results were found to reject the original hypothesis, so the fixed effects model was used for the panel data regression. The panel data of 278 prefecture-level and above cities and 15 eastern cities, 86 central cities, 80 western cities, and 78 eastern cities were used as samples for regression, and the regression results are shown in Table 4.

**Table 4:** The panel data regression results of land finance impacting on economic growth.

**Variables**	**National**	**Eastern region**	**Central region**	**Western region**	**Northeast region**
*landfin*	0.071*** (0.007)	0.074*** (0.021)	0.014*** (0.012)	0.047*** (0.01)	0.038*** (0.012)
*scalegov*	–0.295*** (0.073)	–0.135 (0.188)	–0.173 (0.229)	–0.039 (0.082)	–0.432** (0.184)
*scalehou*	0.029 (0.035)	0.001 (0.048)	0.331*** (0.121)	–0.029 (0.067)	–0.153* (0.078)
*incomeper*	0.641*** (0.064)	0.378*** (0.141)	0.322*** (0.109)	0.558*** (0.129)	0.256** (0.113)
*infras*	–0.046*** (0.009)	–0.041 (0.027)	–0.075*** (0.014)	–0.024* (0.013)	–0.015 (0.013)
*indus*	–0.01 (0.008)	–0.01 (0.008)	1.722*** (0.208)	1.285*** (0.121)	1.817*** (0.119)
*unemploy*	–0.403*** (0.123)	–0.246 (0.357)	–0.112 (0.128)	–0.223 (0.313)	–0.529* (0.306)
Individual effects	Yes	Yes	Yes	Yes	Yes
Time trends	Yes	Yes	Yes	Yes	Yes
_cons	3.707*** (0.635)	6.424*** (1.403)	6.882*** (1.114)	3.824*** (1.25)	6.57*** (1.111)
N	2502	306	774	720	702
R^2^	0.628	0.219	0.727	0.83	0.775

^*^, ^**^, ^***^ indicate significant correlation at the 10, 5, and 1% levels, respectively.

As can be seen from Table 4, the regression coefficients of land finance on urban economic growth are significantly positive at the 1% level for both national cities at the prefecture-level and above, as well as cities in the eastern region, central region, western region, and northeastern region, indicating that land finance has a significant contribution to the economic growth of each city. However, for cities in different regions, there is a large variability in the degree of influence of land finance on economic growth. The economic growth of cities in the eastern region is most affected by land finance with an impact coefficient of 0.074, indicating that each unit increase in land finance causes economic growth to rise by 0.074 percentage points. Economic growth is least affected by land finance in central cities, with an impact coefficient of only 0.014, indicating that each unit increase in land finance drives economic growth up by only 0.014 percentage points. Overall, economic growth in cities at the prefecture-level and above is significantly influenced by land finance, with an impact coefficient of 0.071.

In terms of control and characteristic variables, the significance of regression coefficients varies significantly for different sample subjects. For the overall sample of cities at the prefecture-level and above nationwide, the regression coefficients of control variables and characteristic variables on urban economic growth are significantly correlated at the 1% level, except for the scale of real estate investment and the level of infrastructure construction. At the 10% level, the scale of real estate investment only significantly affects the economic growth of cities in the central region and cities in the eastern region, with a negative coefficient on the economic growth of cities in the northeastern region and a larger positive coefficient of 0.331 on the economic growth of cities in the central region, indicating that for cities in the northeastern region, promoting the real estate sector helps promote regional economic development. at the 5% level, the income level of the population significantly and positively affects the economic growth of all regional cities.

Government size significantly and negatively affects economic growth for both national and northeastern cities, suggesting that the incentive effect of government size is negative, and that reducing government spending and adopting an expansionary fiscal policy is beneficial to regional economic development. The level of industrialization significantly and positively affects the economic growth of cities in the central, western and northeastern regions, indicating that the economic growth of cities in these regions mainly relies on the secondary industry, and more policy support should be given to industrial enterprises to help them improve productivity and thus promote regional economic development.

### Intermediary effect analysis

The effect of land finance on economic growth consists of two parts: direct effect and indirect effect, in which housing price is an important mediating variable in the indirect effect of land finance on economic growth Hausman test results indicate that equation (2) and equation (3) should be estimated using panel fixed effects, and the estimation results are shown in Tables 5, 6.

**Table 5:** The results of land finance impacting on housing prices.

**Variables**	**National**	**Eastern region**	**Central region**	**Western region**	**Northeast region**
*landfin*	0.079^***^ (0.005)	0.05^***^ (0.011)	0.072^***^ (0.01)	0.047^***^ (0.009)	0.060^***^ (0.009)
Individual effects	Yes	Yes	Yes	Yes	Yes
Time Trends	Yes	Yes	Yes	Yes	Yes
_cons	7.949^***^ (0.038)	7.994^***^ (0.081)	8.308^***^ (0.081)	8.06^***^ (0.067)	7.936^***^ (0.063)
N	2502	306	774	720	702
R^2^	0.679	0.332	0.781	0.756	0.679

*** *indicate significant correlation at the 10, 5, and 1% levels, respectively*.

**Table 6:** The mediating effect test results of housing prices.

**Variables**	**National**	**Eastern region**	**Central region**	**Western region**	**Northeast region**
*landfin*	0.056*** (0.007)	0.067*** (0.022)	0.013*** (0.012)	0.045*** (0.01)	0.037*** (0.012)
*prihou*	0.198*** (0.028)	0.181 (0.117)	0.01 (0.045)	0.055 (0.044)	0.018 (0.051)
*scalegov*	–0.291*** (0.072)	–0.156 (0.188)	–0.175 (0.229)	–0.027 (0.082)	–0.435** (0.185)
*scalehou*	0.021 (0.035)	0.003 (0.048)	0.333*** (0.121)	–0.043 (0.068)	–0.152* (0.078)
*incomeper*	0.613*** (0.064)	0.387*** (0.141)	0.321*** (0.109)	0.543*** (0.13)	0.253** (0.114)
*infras*	–0.047*** (0.009)	–0.037 (0.027)	–0.075*** (0.014)	–0.024* (0.013)	–0.015 (0.013)
*indus*	–.009 (0.008)	–0.011 (0.008)	1.726*** (0.209)	1.285*** (0.121)	1.812*** (0.119)
*unemploy*	–0.36*** (0.122)	–0.212 (0.357)	–0.114 (0.128)	–0.241 (0.313)	–0.531* (0.307)
Individual effects	Yes	Yes	Yes	Yes	Yes
Time trends	Yes	Yes	Yes	Yes	Yes
_cons	2.4*** (0.655)	4.877*** (1.721)	6.963*** (1.173)	3.527*** (1.271)	6.462*** (1.153)
N	2502	306	774	720	702
R^2^	0.636	0.226	0.727	0.83	0.775

^*^, ^**^, ^***^ indicate significant correlation at the 10, 5, and 1% levels, respectively.

The results in Table 5 show that for all regional cities, land finance significantly and positively affects the mediating variable housing price, but there is variability in the degree of influence. On the one hand, it shows that urban housing prices are related to land supply and land cost is an important component of housing prices; on the other hand, it shows that there is regional variability in the impact of the land market on housing prices. This variability may be related to the control of land by local governments. The western region was the first to adopt the pilot linkage of construction land increase and decrease, so the impact of land finance on housing prices is smaller.

The results in Table 6 show that with the inclusion of housing price as a mediating variable, land finance still has a significant contribution to economic growth at the 5% level, but the mediating variable housing price only has a significant contribution to economic growth in the national sample cities, while it does not have a significant effect on economic growth in the eastern, central, western and northeastern regions. According to the new procedure of mediating effect test, the above five types of cities need to be tested separately. For the national sample cities, Because the regression coefficients α_1_, β_1_, γ_1_ and γ_2_ are significant and β_1_ × γ_2_ and γ_1_ have the same sign, there is a partial mediating effect of housing prices on land finance affecting economic growth.

That is, land finance partially affects economic growth in the national sample cities through housing prices. For the eastern, central, western and northeastern regions, although the regression coefficients α_1_ and are significant, γ_2_ is not significant, so Bootstrap method needs to be used to test whether β_1_ × γ_2_ is significant. Randomly conducted 5,000 times, the results of bootstrap test for housing prices in the eastern, central and western regions include 0, indicating that the mediating effect of housing prices on economic growth is not significant. The results of the bootstrap test for housing prices in the Northeast region do not include 0, indicating that the mediating effect of housing prices on economic growth is significant (see Table 7 for specific regression coefficients and confidence intervals). Regarding the control and characteristic variables, the significance of the regression coefficients of the control and characteristic variables affecting economic growth did not change after the inclusion of the mediating variable of housing price, and the direction of the effect did not change, with a slight increase or decrease in the degree of influence.

**Table 7:** The bootstrap test results.

**City type**	**Regression coefficient**	**Confidence interval**	**Test results**
Eastern region	0.0183	[−0.0363, 0.0730]	Including 0, failed the test
Central region	0.0050	[−0.0040, 0.0141]	Including 0, failed the test
Western region	−0.0109	[−0.0011, 0.0244]	Including 0, failed the test
Northeast region	0.0207	[0.0037, 0.0377]	Excluding 0, passing the test

In summary, the paths and extent to which land finance affects economic growth through housing prices in the national sample cities and the eastern, central, western, and northeastern regions are shown in Table 8.

**Table 8:** The path test result of land finance impacting on economic growth.

**City type**	**Total effect**	**Direct effect**	**Indirect effects**
National	0.071	0.056	0.015
Eastern region	0.074	0.067	Not significant
Central region	0.014	0.013	Not significant
Western region	0.047	0.045	Not significant
Northeast region	0.038	0.037	0.001

From the results in Table 8, it can be seen that there is a significant mediating effect of housing prices on the economic growth of land finance in the national sample cities and northeastern cities, accounting for 22.03 and 2.84% respectively, while the mediating effect of housing prices on the economic growth of land finance in the eastern, central and western cities is 0. The reason may be that the real estate market in these three types of cities is overheated, attracting industrial capital to idle in the real estate market, which inhibits industrial transformation and upgrading and eventually undermines the potential of economic growth. The reason may be that the real estate market in these three cities is overheated, attracting industrial capital to idle in the real estate market, resulting in the “de-realization of capital” and “drifting away from reality,” inhibiting industrial transformation and upgrading, and hollowing out the industry in Germany, which ultimately undermines the economic growth potential.

### Robustness tests

To test the robustness of the findings, the model is regressed stepwise again by replacing the core variables. The explanatory variable land finance is lagged by one period to further avoid endogeneity problems, i.e., the instrumental variable is ”lagged term of land finance.” GDP growth rate is used to replace GDP per capita as the proxy variable for economic growth, and the average price of second-hand house sales is replaced by the average price of urban housing sales as the proxy variable for housing prices by taking the logarithm. Other variables were kept unchanged. The regression results after replacing the variables are similar to the results of previous studies, thus determining the robustness of the results of this study.

## Conclusion and policy implications

The random-effects model of land finance affecting economic growth is constructed by taking national prefecture-level and above cities as research objects, theoretically analyzing the effects of land finance on housing prices and economic growth as well as the effects of housing prices on economic growth and considering the mediating role of housing prices. 278 prefecture-level and above cities with relatively well-developed land and real estate markets were selected to test the mediating effect of urban housing prices on land finance affecting economic growth by national sample cities and different regional sample cities respectively based on panel data from 2011–2019. The following studies were conducted and findings were drawn.

1. The impact of land finance on urban economic growth. Land finance has a significant contribution to the economic growth of the cities in the national sample and four different regions, but there is some variability in the degree of influence. The economic growth of cities in the eastern region is most influenced by land finance, with an impact coefficient of 0.074; the economic growth of cities in the central region is least influenced by land finance, with an impact coefficient of 0.014.
2. The effect of land finance on urban housing prices. For both the national sample cities and the four different regional cities, land finance significantly and positively affects housing prices, but there is some variability in the degree of influence. Housing prices in the central region are most affected by land finance with a coefficient of 0.072, while housing prices in the western region are least affected by land finance with a coefficient of 0.047.
3. Mediating effect of housing prices on land finance affecting economic growth. There is a significant partial mediating effect of housing prices on land finance affecting economic growth in the national sample cities and cities in the northeast region, with 22.03% and 2.84%, respectively. The mediating effect of urban housing prices on land finance affecting economic growth in the eastern, central, and western regions is not significant. The mediating effect can be identified as 0.
4. The effects of control and characteristic variables on economic growth. The significance of the regression coefficients of the control and characteristic variables affecting economic growth did not change before and after the inclusion of housing price mediating variables, nor did the direction of influence change, with a slight increase or decrease in the degree of influence.

### Based on the above findings, the following recommendations are made

1. To formulate land finance policies based on city-specific policies and further give full play to the positive role of land finance. From the empirical findings, we can see that land finance has a significant role in promoting urban economic growth, so we should continue to promote land market reform, play the role of the market in land resource allocation, improve the efficiency of land resource use, and give full play to the positive role of land finance. Different programs should be adopted for different regional cities to optimize land finance revenue methods and expenditure structures. For the eastern region, we should implement the policy of linking people and land, reasonably determine the land price of commercial housing, ensure the stability of local government revenue, and reasonably guide land finance to support infrastructure construction and improvement. For the central, western, and northeastern regions, the local government taxation system should be further improved to enhance the sustainability of local government revenue and coordinate local government expenditure paths.
2. Further guide to enhance the balanced development of real estate and the real economy. The real estate industry has been an important engine of economic growth due to its many related industries and long industrial chain. However, its virtual characteristics can also attract a large amount of financial capital to idle in the industry, leading to a “de-realization” and “de-realization,” especially in the development stage of industrial restructuring, transformation and upgrading. Therefore, on the one hand, the government should effectively guide the reasonable expectations and demand for commercial housing purchases, boost consumer confidence in the real estate market, give full play to the role of market regulation, promote supply-side reform of the real estate market, and promote the stable operation of housing prices and the stable and healthy development of the real estate market. On the other hand, it should pay fundamental attention to the development of the real economy, reform the business environment in terms of financial support, land supply, tax incentives and other aspects of comprehensive reform, and take advantage of land resources to improve infrastructure, control the virtual economy, guide the flow of capital to the real economy, and promote industrial transformation and upgrading.

This paper empirically tests the mediating effect of housing prices on economic growth influenced by land finance in prefecture-level and above cities in China, further enriches the study of the interrelationship between land, housing and macroeconomics at the national scale, and also provides empirical evidence for exploring the path of urban regional economic development, but there are some shortcomings in the study: first, due to the limitations of the degree of housing market development and the difficulty of obtaining monitoring data, the study sample does not cover all resource-based cities, and the study period is only 2011–2019, the time scale is not long enough; secondly, the variables land finance, housing price and economic growth have a strong locational dimension, and the spatial factor is also a key element to be considered from the Spatio-temporal dimension, while the model construction does not consider the spatial element to introduce spatial weights. In the follow-up, we will calculate the spatial weight between cities and build a spatial econometric model to analyze how housing prices and land finance interact to affect economic growth considering spatial utility.

## Data availability statement

The original contributions presented in the study are included in the article/supplementary materials, further inquiries can be directed to the corresponding author/s.

## Ethics statement

The studies involving human participants were reviewed and approved by Hunan City University Institutional Review Board. The participants provided their written informed consent to participate in this study.

## Author contributions

QS, YF, and YT contributed to conception and design of the study, performed statistical analysis, and wrote the first draft of the manuscript. SJ collected the data, contributed to manuscript revision, read, and approved the submitted version. All authors contributed to the article and approved the submitted version.

## Funding

This work was supported by grants from the Provincial Natural Science Foundation of Hunan (2022JJ30116), Think Tank Special Project of Social Science Fund of Hunan Province (19ZWB63), Base Project of Social Science Fund of Hunan Province (21JD041), and Excellent Youth Fund of Hunan Provincial Department of Education (18B440).

## Conflict of interest

The authors declare that the research was conducted in the absence of any commercial or financial relationships that could be construed as a potential conflict of interest.

## Publisher's note

All claims expressed in this article are solely those of the authors and do not necessarily represent those of their affiliated organizations, or those of the publisher, the editors and the reviewers. Any product that may be evaluated in this article, or claim that may be made by its manufacturer, is not guaranteed or endorsed by the publisher.
